# FALL RISK FACTOR SCREENING AND MANAGEMENT USING VIRTUAL PLATFORMS, PREVALENCE, AND PERCEPTIONS

**DOI:** 10.1093/geroni/igae098.0707

**Published:** 2024-12-31

**Authors:** Alexander Garbin

**Affiliations:** VA Eastern Colorado Healthcare System, Aurora, Colorado, United States

## Abstract

Numerous barriers limit comprehensive assessment and management of fall risk factors within primary care including limited time and competing medical priorities. Virtual platforms provide an opportunity outside of primary care to remotely screen fall risk factors and deliver fall prevention interventions. However, researchers and clinicians may be hesitant to assess and intervene on multifactorial fall risk factors through virtual platforms due to perceived safety concerns, difficulty adapting outcome measures to a virtual environment, and potential technological challenges. This presentation will detail strategies and results from a study examining the prevalence of falls, fall risk, and multifactorial fall risk factors in older Veterans seen within Veterans Health Administration (VHA) primary care using a virtual platform (VA Video Connect). Fall risk factor measurements discussed will include the 5 Times Sit to Stand, the Survey of Activities and Fear of Falling in the Elderly, the Home Falls and Accidents Screening Tool, and the Screening Tool of Older Persons Prescriptions in older adults with high fall risk. Results from semi-structured interviews with Veterans and VHA primary care providers will also be discussed with an emphasis on Veterans’ and providers’ experiences with fall risk factor assessment and management within VHA primary care as well as their perceptions of using telehealth for identifying and managing fall risk factors. Together, this presentation will provide insight on the potential for using virtual platforms to assess multifactorial fall risk factors as well as the perceived barriers and facilitators of identifying and managing fall risk factors in a virtual setting.

